# Validation of a Novel Commercial ELISA Test for the Detection of Antibodies against *Coxiella burnetii*

**DOI:** 10.3390/pathogens9121075

**Published:** 2020-12-21

**Authors:** Salvatore Ledda, Cinzia Santucciu, Valentina Chisu, Giovanna Masala

**Affiliations:** Zoonotic Pathology and OIE Reference Laboratory for Echinococcosis, National Reference Center for Echinococcosis (CeNRE), IZS della Sardegna, 07100 Sassari, Italy; salvatoreledda.mail@tiscali.it (S.L.); valentina.chisu@izs-sardegna.it (V.C.); giovanna.masala@izs-sardegna.it (G.M.)

**Keywords:** *Coxiella burnetii*, Q fever, immunological diagnosis, human diagnosis, validation, quality management, ISO/IEC 17043, ISO/IEC 17025

## Abstract

Q fever is a zoonosis caused by *Coxiella burnetii*, a Gram-negative pathogen with a complex life cycle and a high impact on public and animal health all over the world. The symptoms are indistinguishable from those belonging to other diseases, and the disease could be symptomless. For these reasons, reliable laboratory tests are essential for an accurate diagnosis. The aim of this study was to validate a novel enzyme-linked immunosorbent assay (ELISA) test, named the Chorus Q Fever Phase II IgG and IgM Kit (DIESSE, Diagnostica Senese S.p.A), which is performed by an instrument named Chorus, a new device in medical diagnostics. This diagnostic test is employed for the detection of antibodies against *C. burnetii* Phase II antigens in acute disease. Our validation protocol was performed according to the Italian Accreditation Body (ACCREDIA) (Regulation UNI CEI EN ISO/IEC 17025:2018 and 17043:2010), OIE (World Organization for Animal Health), and Statement for Reporting Studies of Diagnostic Accuracy (STARD). Operator performance was evaluated along with the analytical specificity and sensitivity (ASp and ASe) and diagnostic accuracy of the kit, with parameters such as diagnostic specificity and sensitivity (DSp and DSe) and positive and negative predictive values (PPV and NPV), in addition to the repeatability. According to the evaluated parameters, the diagnostic ELISA test was shown to be suitable for validation and commercialization as a screening method in human sera and a valid support for clinical diagnostics.

## 1. Introduction

Q fever is a disease caused by *Coxiella burnetii*, a ubiquitous, zoonotic, Gram-negative bacterium isolated from a wide variety of animals, arthropods, and environmental samples, with a worldwide distribution, except for New Zealand [1]. Recently, the axenic cultivation of *C. burnetii* has led to this pathogen no longer being considered an obligate intracellular bacterium [2,3].

*C. burnetii* exists in two distinct morphological variants, a small-cell variant (SCV) and a large-cell variant (LCV), corresponding, respectively, to the metabolically inactive and replicative forms of *C. burnetii* in the environment and in the cell hosts, respectively [4]. *C. burnetii* can be found in two phases, called Phase I and Phase II, that are linked to the antigenic variations of its lipopolysaccharide (LPS). Phase I represents the natural phase, which is highly infectious with a smooth LPS, while Phase II is obtained *in vitro* after *C. burnetii* propagation in cell culture; it is non-infective, with a rough-truncated LPS and a different sugar composition compared to the virulent form [1,5].

Domestic mammals, mainly goats, sheep, and cattle, represent the most important reservoir of infection of this bacterium [6]. However, wild vertebrates are also considered as putative reservoirs of Q fever [5]. Transmission from animal reservoirs to humans occurs primarily through the inhalation of contaminated aerosols. However, other modes of transmission, such as human-to-human infection [6,7,8], the ingestion of contaminated animal products or even tick bites [9], have also been demonstrated [6,10,11,12].

Q fever has a wide spectrum of clinical manifestations, ranging from asymptomatic or mildly symptomatic seroconversion to hepatitis or severe pneumonia. The acute infection is often self-limiting, and clinical signs include fever, headache, myalgia, muscle cramps, and respiratory complications [13]. Furthermore, in 2–5% of acute cases, it can result in a chronic form, frequently causing endocarditis or vascular infection [1,14]. Very often, symptoms are indistinguishable from those belonging to other diseases; for this reason, laboratory tests are essential for an accurate diagnosis [15]. 

Between 2009–2013, in Europe, 648 cases of Q fever were reported, with a notification rate of 0.17 cases/100,000 inhabitants, while in Italy, the hospitalization incidence rate corresponded to 0.55 cases/1,000,000 hospitalized subjects. However, a notable variability was highlighted in the distribution among the regions: Sassari in Sardinia (Italy) showed the highest average rate for this five-years period, equal to 11.9 cases/1,000,000 hospitalized subjects [16]. In Sardinia, *C. burnetii* is a notifiable disease that is responsible for abortion and stillbirth in small ruminants and thus plays an important role in the economy of the island by causing huge losses for breeders [17]. Since ruminants are recognized as the main source of infection in humans, identifying *C. burnetii* in a fast, sensible, specific, and cheap way represents a global challenge.

Serological tests are the most common tools for testing Q fever; they include the complement fixation test (CFT), the indirect immunofluorescent antibody test (IFAT) and the enzyme-linked immunosorbent assay (ELISA). IFAT is the gold standard for the laboratory detection of Phase I and Phase II antibodies against *C. burnetii*. Anti-phase II antibodies are predominant during primary infection and detectable up to 15 days after the onset of clinical symptoms; moreover, their IgG titer tends to be higher than that of Phase I [1,13,18]. Moreover, anti-phase II antibodies are predominant in acute Q fever, and IgMs are the first antibodies to be detected in blood, followed by IgG Phase II. However, a detectable response of antibodies could be noted after 2 weeks, with titers decreasing within 3–6 months. The diagnosis of acute Q fever is based on seroconversion or a four-fold increase in IgG and IgM antibody titers between two sera taken 3–6 weeks apart [18].

The diagnosis of acute infection by IFAT can display differences between laboratories and countries, although it can usually be performed considering titers of Phase II IgG ≥ 1:200 and/or IgM ≥ 1:50 as cut-offs [13,19]. Despite IFAT and CFT being frequently used, they show several limitations; for example, they are not standardized among laboratories, and consequently the interpretation of their results may vary, and they do not lend themselves to large-scale screening, since they cannot be automated [20]. These disadvantages emphasize the need to develop new, standardized tests for the screening of *C. burnetii* infections.

Immunological tests also need to have high sensitivity and specificity to be able to detect molecule targets in the diagnostic field. In particular, cross-reactions against several pathogens, such as *Legionella* spp., *Chlamydia* spp., *Bartonella* spp., *Bordetella* spp., *Brucella* spp., and also *Mycoplasma pneumonie*, have been described by several authors [21,22,23,24,25,26,27,28].

Difficulties in isolating *C. burnetii* from infected samples, the hazard for laboratory operators, the high variability of the clinical manifestations and the lack of clear pathognomonic features make the diagnosis of Q fever challenging [29]. The use of reliable and validated measurement instruments is of critical importance for the analysis of results and clinical practice.

The aim of our study was to validate a new diagnostic kit to be used for serological screening as a support for clinical diagnostics in human patients affected by Q fever. The validation plan of this study was designed considering all the parameters already evaluated by the manufacturing company. Concerning specificity, several cross-reactions for same pathogens such as *Legionella* spp., *Brucella* spp., *Bordetella* spp., and *Mycoplasma pneumonie* have already been carried out. For this purpose, we decided to evaluate the specificity of the test using human sera positive for *Chlamydia* spp., *Bartonella* spp., and *Rickettsia* spp. [21,22,23,24,25,26,27,28].

Our validation protocol was performed according to the Italian Accreditation Body (ACCREDIA) (Regulation UNI CEI EN ISO/IEC 17025:2018 and 17043:2010) [30,31], OIE (World Organization for Animal Health) [32], and Statement for Reporting Studies of Diagnostic Accuracy (STARD) [33,34].

Operator performance was evaluated along with the analytical specificity and sensitivity (ASp and ASe) and diagnostic accuracy of the kit, such as diagnostic specificity and sensitivity (DSp and DSe) and positive and negative predictive values (PPV and NPV).

According to the findings of the evaluated parameters, the novel diagnostic ELISA test was shown to be suitable for validation and commercialization and a valid support for the clinical diagnosis, improving the surveillance of patients at risk of contracting Q fever.

## 2. Results

### 2.1. Operator Performance

In this study a novel ELISA test, able to detect IgG and IgM against *C. burnetii*, was evaluated. In order to determine the capability of the laboratory to perform the diagnostic assay and produce reliable results, the operator performances have to be evaluated. For this reason, an operator followed a practical training course, then a proficiency test. 

#### Accuracy and Repeatability

The intraoperator repeatability, concordance degree detected by comparing different replicates of results, obtained by Chorus, along with the accuracy, agreement of the results obtained by Chorus and compared to the real value equal to the clinical and IFAT findings, were determined. Agreement among results was assessed by Cohen k coefficient (κ), calculated according to Byrt table [35].

Two blind sets of 20 samples were prepared by an operator external to the circuit to be analyzed by Chorus Q fever IgG and IgM kits, respectively (Table 1). The comparison between the results obtained by the operator following the duplicate analysis of the same set of 20 samples by the Chorus Q Fever Phase II IgG assay (Table 1) presented a high concordance, as the intraoperator repeatability value was excellent (Cohen’s K = 1) (Table 2). Moreover, following the comparison of the findings of the operator, by means of Chorus Q Fever IgG along with those of the clinical findings and the Human Q Fever IFA IgG Antibody Kit (IFAT) (Fuller Laboratories, Fullerton, CA, USA), a very good accuracy value was found (Cohen’s K = 0.89) (Table 2).

Concerning the operator performances evaluation on the set of 20 samples (Table 1) by the Chorus Q Fever Phase II IgM test, for the agreement between the results for the intraoperator repeatability was very good (Cohen’s K = 0.88) (Table 2). While for the accuracy, the coefficient value was excellent (Cohen’s K = 0.94) (Table 2).

### 2.2. Analytical Performance of the Assay

The assay validation needs to establish the analytical sensitivity (the smallest detectable amount of a analyte target), as well as the analytical specificity (the degree to which the test does not cross-react with any analyte associated with other infections). These parameters are necessary to quantify the level of detection (ASe) and the capacity to capture the target analyte (Asp), respectively.

#### 2.2.1. Analytical Sensitivity

The evaluation of ASe was determined by analyzing two sets of serial dilutions, calculating the limit of detection (LOD) that correspond to the previous dilution point in which the analyte was no longer distinguishable from the control matrix. The results obtained by the novel test, the Chorus Q Fever Phase II IgG and IgM, were compared to those obtained by IFAT, the gold standard test.

The analyzed duplicate serial dilutions showed that the first OD negative signal detected by the Chorus Q Fever Phase II IgG corresponded to the 1:16 dilution, that is two dilution points less than those resulted by IFAT, in which the first negative signal was at 1:64. This dilution point of 1:16 was equivalent to a concentration of total proteins greater than 2.0 µg/mL, while the 1:64 corresponded to 1.1 µg/mL (Table 3).

While, for the Chorus Q Fever Phase II IgM kit, the first OD negative signal corresponded to a concentration of 1:4 (greater than 2.0 µg/mL of total proteins), as well as for those resulted by IFAT (Table 3). Total proteins were quantified by a Pierce™ BCA Protein-Assay-Kit (Thermo Fisher Scientific™, Waltham, MA, USA; 23225).

#### 2.2.2. Analytical Specificity

In order to evaluate the capability of the novel Chorus Q Fever test to specifically detect the target analyte, a panel of sera from patients affected by pathogens different from *C. burnetii* were analyzed.

Specifically, ASp was assessed following the determination of the cross-reactivity profile with a panel of five groups of 66 sera analyzed by means of the Chorus Q Fever Phase II IgG and IgM kits. The set comprised control samples positive for *C. burnetii* (IgG and IgM), negative controls and 46 other samples from patients negative for *C. burnetii* but serologically positive for *Bartonella* spp., *Rickettsia* spp. and *Chlamydia* spp. The results of each serum, displayed in Table 4, were entered into a 2 × 2 contingency table (not shown). The ASp values were equal to 100% for IgG and 96% for IgM, respectively. Since it was not possible to include doubtful samples in the Excel spreadsheet, cross-reactivity was detected only for one sample belonging to *Chlamydia* spp. in the test for the IgM; conversely, in *Bartonella* spp. and *Rickettsia* spp. sera, no false positives for IgG or IgM were detected (Table 4).

#### 2.2.3. Diagnostic Accuracy: Diagnostic Sensitivity, Diagnostic Specificity, Positive Predictive Value and Negative Predictive Value

A useful way to determine the DSe, DSp, PPV and NPV is by means of a contingency table (2 × 2), considering the known infection status and the negativity of the samples. Positive serum samples from patients with the symptoms related to Q fever and with a positive titer for antibodies versus *C. burnetii* (IgG and IgM) by IFAT were classified as true positive, whereas all sera from patients without any symptoms related to Q fever and that resulted negative to IFAT were considered as true negatives. Consequently, all samples that in the analysis by Chorus presented an antibody response were considered positive only if they belonged to the category of true positives and were considered negative if they were from a healthy patient. Following the analysis of the total of 340 sera by Chorus Q fever Phase II for both IgG and IgM kits, the results obtained for the IgG kit showed, out of 101 true positive sera tested, 98 positive and three negative samples. In addition, out of 77 true negative sera tested, 71 resulted negative, two were positive, and four were doubtful. According to these data, DSe and DSp showed values of 97% and 92%, respectively (Table 5).

Moreover, concerning the IgM kit, out of 63 true positive sera tested, 60 resulted positive and three were negative, while out of 99 true negative sera analyzed, 93 were negative, two were positive and four were doubtful. DSe and DSp showed values of 95% and 94%, respectively (Table 6). Data on the DSe and DSp related to the disease in the target population made it possible to calculate the PPV and NPV of the assay in the same contingency table. According to our results, PPVs and NPVs for the IgG kit were 94% and 96%, respectively, (Table 5); finally, PPVs and NPVs for the IgM kit were 91% and 97%, respectively, (Table 6).

## 3. Discussion

In this work, we evaluate the analytical and diagnostic performances of a novel device in medical diagnostics, the Chorus Q Fever Phase II IgG and IgM Kit (DIESSE, Diagnostica Senese S.p.A), to verify if this method is suitable for validation. To this end, diagnostic and analytical parameters regarding the accuracy, and repeatability of the novel test of the Chorus Q Fever Phase II IgG and IgM kit were evaluated; moreover, the test results were compared to the clinical findings and IFAT, as a gold-standard assay.

Although IFAT is the current reference method for serological diagnosis, it has numerous disadvantages such as the subjective judgment of fluorescence, as well as the cost and the execution time of the test [36]. Moreover, IFAT requires time and dedicated expert operators. On the other hand, the Chorus system is automated and easy to perform with reduced analysis times, 30 tests can be performed simultaneously. All the reagents contained in the device are ready to use, and there is no need for titration and/or dilutions, which represent the most common sources of error. Finally, spectrophotometric reading and data processing through dedicated software eliminate subjectivity in the interpretation of the analytical results, which allows a standardization of the method. However, IFAT results in a more sensitive and specific method.

Thus, among the multiple aspects that are usually considered in the validation process of a diagnostic test, the repeatability have to be evaluated [37]. According to the Regulation UNI CEI EN ISO/IEC 17043 [31], our results related to the operator performances demonstrated excellent and very good values of the Cohen’s κ coefficients for repeatability and accuracy for both kits (Table 2). Moreover, the accuracy and repeatability, of the method also showed very good values of the Cohen’s κ coefficients.

The estimation of ASe confirmed the higher sensitivity of the IFAT respect to Chorus, as reported by other authors [20]. As suggested by the OIE manual [32], we firstly determined the limit of detection (LOD); however, it was not possible to conduct a direct quantification of the analytes (antibodies against *C. burnetii*) between the two techniques, and consequently we compared the OD obtained by Chorus to those from IFAT for each point of the serial dilutions, after the total protein quantification of each dilution point [9,32]. The results also showed a difference of two dilution points between IFAT and the Chorus Q Fever Phase II-IgG and a difference of one dilution point between the IFAT and the Chorus Q Fever Phase II-IgM (Table 3). On the other hand, these results also demonstrated a good sensitivity for the Chorus kit, for IgG and even higher sensitivity for IgM. More precise data could be obtained following the analysis of a greater number of sera.

Concerning the ASp, we obtained very good and remarkable results (Table 4). As determined by the contingency table, the acquired values were equal to 100% for IgG and 96% for IgM, as cross-reactivity was not observed following the analysis by this novel Chorus device of the sera from patients affected by *Bartonella* spp. and *Rickettsia* spp. for both IgM and IgG antibodies. Conversely, cross-reactivity was only detected in the reaction with *Chlamydia* spp. for the sera tested for Q fever Phase II IgM; nevertheless, three sera for Phase II IgM and one serum for Phase II IgG gave doubtful results, but it was not possible to include doubtful outcomes in the Excel spreadsheet. However, a low specificity in discriminating *Chlamydia* spp. antibodies has been reported by other authors [23,24,27,28], indicating that these results may need more specific diagnostic tools. However, false-positive results could be presented by the Chorus Q fever kit versus the antibodies of *Legionella* spp., *Brucella spp.*, *Bordetella spp.*, and *Mycoplasma pneumonie,* as reported by the kit’s datasheet. This data has been confirmed by several studies involving immunological diagnostic tests [21,22,26].

An ideal diagnostic test must correctly discriminate sick from healthy patients, and once this is performed, individuals have to be classified with absolute certainty as affected or unaffected by the disease of interest. A method with a high sensitivity will provide an accurate proportion of positivity among true positives; additionally, a more specific method will result in a higher proportion of negativity among the real negatives. Consequently, in daily practice, the PPV and NPV are very important as they guarantee the probability of having the disease after a positive test result (PPV) and the probability of not having the disease after a negative test result (NPV) [37]. This ELISA test performed by the Chorus system showed excellent performance especially in terms of DSe (97% for IgG, 95% for IgM) and NPV (96% for IgG, 97% for IgM) (Table 5 and Table 6). The parameters related to DSp (92% for IgG, 94% for IgM) and PPV (94% for IgG, 91% for IgM) (Table 5 and Table 6) were characterized by lower values due to the cross-reactivity detected versus *Chlamydia* positive samples. Nevertheless, according to the expected values for the validation of immunological tests, our results are consistent.

Several studies have been carried out on increasing the sensitivity and specificity of immunological tests for antibodies against *C. burnetii* by using a combination of specific proteins—either recombinant or derivatives—directly from *Coxiella*. In particular, several chaperones, such as the outer membrane chaperone OmpH, the heat shock protein GroEL, the peptidyl-prolyl cis-trans isomerase Mip, the surface protein YbgF and the *Coxiella* outer membrane protein Com1, were described as immunoreactive proteins [38,39,40,41,42,43]. Our data on sensitivities and specificities confirmed previously published results on studies in veterinary medicine on animal sera (sheep, goats, and cattle). By means of this home-made ELISA functionalized by the recombinant protein Com1, sensitivities and specificities reported rates corresponding to 71–94% and 68–77%, respectively [44]. Moreover, our values of specificities and sensitivities were shown to be higher with respect to those obtained in another study on Com1 as an antigen used for human sera, corresponding, respectively, to 71% and 47% [45].

Difficulties in differentiating between uninfected and infected patients are seldom reported by serological tests, especially in the early stages of infectious diseases. More specific diagnostic tools may also help obtain accurate clinical diagnosis. Identifying the exact moment of the symptom’s onset will lead to the choice of the most suitable screening test and a subsequent treatment, which will lead to a better outcome. Laboratory diagnosis of acute Q fever is ideally based on a combination of clinical examination, serology analysis and polymerase chain reaction (PCR) [5]. The use of the PCR could guarantee an early diagnosis, since this technique is able to detect *C. burnetii* DNA in the whole blood within 2 weeks from the onset of symptoms [46] if the patient had not previously undergone a pharmacological treatment with antibiotics [47]. PCR is also helpful to guide treatment decisions; it was even considered indispensable in a diagnostic protocol that was recently applied in a Q fever epidemic in the Netherlands [48].

## 4. Conclusions

The results concerning the evaluation of all parameters evidenced excellent values and demonstrated that the novel Chorus Q fever ELISA is a robust assay for detecting *C. burnetii* antibodies during the acute phase of the disease. Consequently, this method is suitable for validation and could also be commercialized. This test could represent a valid support for clinical diagnostics, improving the surveillance of patients at risk of contracting Q fever.

## 5. Materials and Methods

### 5.1. Sampling

A total of 340 serum samples were included in the study: 164 belonged to patients clinically affected by acute Q fever, while 176 sera were from healthy donors and from patients infected by other parasites, in both cases without any symptoms attributable to Q fever. The former group employed for this validation protocol was representative of the positive sample set; for this purpose, it was decided to use sera from clinically affected Q fever patients that had also been tested positive by IFAT. The latter group represented the negative control sample set.

Firstly, blood samples were taken into a sterile tube without any anticoagulant. If clotting was not completed, samples were centrifuged at 250× *g* for 10 min and, subsequently, sera were promptly analyzed or frozen at −20 °C before being examined. All the samples were analyzed by IFAT—the conventional routine method used for the screening samples in our laboratory. Diagnostic criteria were a seroconversion or a two-fold or higher increase in anti-phase II antigen IgG and IgM antibody titers.

For this purpose, all samples included in the study were tested by the novel Chorus Q Fever Phase II IgG-IgM Kits. In particular, the 164 positive samples were constituted by 101 sera analyzed for IgG and 63 sera examined for IgM, along with the remaining 176 samples, used as negative controls, represented by 77 samples for IgG and 99 for IgM. In order to evaluate cross-reactivity with other intracellular pathogens, a total of 46 sera belonging to the negative group from patients with serologically confirmed infections other than Q fever were tested. Specifically, they comprised sera from patients with infections due to *Chlamydia* spp. (*n* = 21), *Rickettsia* spp. (*n* = 10), and *Bartonella* spp. (*n* = 15).

All specimens used for the described analysis were from the sera bank stored at −80 °C at the Zoonotic Pathology Laboratory, at the Istituto Zooprofilattico of Sardinia, Italy.

### 5.2. Ethical Statement

The management of the patient’s data and all procedures related to the human samples of this study were performed in agreement with the rules of ethical standards of the Declaration of Helsinki of 1975, revised in 2013. Additionally, the ethics committee of the Local Health Authority of Sassari (Protocol n° 1136) approved investigation of human samples by the IZS della Sardegna, as required by the National Health Service since 26 March 2013. Written informed consent was obtained from all patients or patients’ parents, depending on age.

### 5.3. Validation Plan

The validation plan applied in this study followed the approach suggested by the World Organization for Animal Health (OIE), the Italian Certification Body (ACCREDIA), our laboratory guidelines, the Standards for Reporting Diagnostic Accuracy (STARD), and finally “The ELISA guide book” [30,31,32,33,34,49].

According to the guidelines listed above, to validate the Chorus Q Fever Phase II IgG and IgM kits, the diagnostic accuracy and analytical performance of the assay and the operator performance were evaluated and compared with a gold standard test, the IFAT. In particular, the parameters ASe, ASp, DSe and DSp, PPV and NPV were assessed, which are the most important indicators of the method performance, as they must be established to evaluate the method’s ability to provide accurate results for sick and healthy patients [32,50]. Finally, concerning the operator performance, accuracy and repeatability were evaluated.

According to our protocols, the validation plan was drawn up by an operator external to the circuit, who also dealt with the preparation of all the different sets of samples and the succession of the sample analyzed.

### 5.4. Indirect Fluorescent Antibody Test for Differential Diagnosis of C. Burnetii Infections

A commercial “Human Q Fever IFA IgG Antibody Kit” was used to detect anti-*C. burnetii* antibodies. IFAT was performed according to the manufacturer’s instructions with the exception of the first dilution step: serum samples were diluted four-fold, and a titer ≥ 1:50, as first dilution point, was considered positive for both IgG and IgM against Phase II. Slides containing the samples were read using a fluorescence microscope with 400× magnification and channel framework for fluorescein isothiocyanate (FITC) (with the most extreme excitation wavelength being 490 nm, and a mean outflow wavelength of 530 nm).

### 5.5. Enzyme-Linked Immunosorbent Assay Applied to Chorus Instrument

The tests used in the present validation study are the Chorus Q Fever Phase II IgG and IgM Kits, based on the ELISA principle, which are performed by the Chorus instrument (Diesse Diagnostica Senese S.p.A, Monteriggioni, Italy), which is a new device in medical diagnostics [51,52,53,54]. The Chorus system includes an automated processor and a diagnostic kit, which consists of monotest strips with seven microplate wells, each covered with a labeled foil seal. Every strip, coated with Phase II *C. burnetii* antigens, contains the conjugate anti-human IgG or IgM monoclonal antibodies, labeled with horseradish peroxidase in phosphate buffer containing phenol 0.05%, Bronidox 0.02% and all the reagents to perform the test by the Chorus instrument. The assay was performed according to the manufacturer’s instructions: before starting the test, sera, devices, washing and cleaning solutions were brought to room temperature and the hydraulic and optical checks were performed; 50 μL of undiluted serum were dispensed in the well of each device dedicated to the sample, and a device for the cut-off calibrator was used for each batch; all the devices with a label comprising a bar code which indicated the assay code, sample code, kit lot number, and expiration date were placed in the Chorus instrument (up to 30 tests can be performed simultaneously); the test and the calibration were performed as reported in the Instrument Operating Manual. A control serum was used to check the validity of the results obtained (if the instrument signals of the control serum showed a value outside the acceptable range, the calibration needed to be repeated, and the previous result was automatically corrected).

The Chorus instrument expressed the result (visible in the instrument display) in optical density (OD) measured at 450 nm as the value index calculated using the formula OD sample/OD cut-off. Concerning the interpretation of results, a positive sample was defined as having a sample absorbance/calibrator absorbance ratio >1.2, a negative sample had a ratio <0.8, and a doubtful sample had a ratio value between 0.8 and 1.2.

### 5.6. Operator Performance: Accuracy and Repeatability

To evaluate the performance of the operator, the accuracy and repeatability were estimated. For this purpose, according with the OIE and ACCREDIA guidelines [30,31,32,33,34] as well as those of our laboratory, a blind set of 40 serum samples (20 for IgG and 20 for IgM) was analyzed by the same operator in duplicate by the Chorus Q Fever Phase II IgG and IgM kits, independently for each test, in the same laboratory and with the same batch to determine the repeatability. Moreover, the accuracy was assessed after the evaluation of the operator’s results obtained by Chorus with those gained by IFAT.

The obtained results were entered into specific Excel modules used to determine the concordance degree among the results for both accuracy and repeatability, expressed by means of the Cohen k coefficient (κ), calculated according to Byrt table [35].

### 5.7. Analytical Performance of the Assay

#### 5.7.1. Analytical Sensitivity

ASe was determined by calculating the limit of detection (LOD), such as the previous dilution point in which the analyte was no longer distinguishable from the control matrix, and comparing the serial dilution sets analyzed, respectively, by IFAT and Chorus. For this purpose, two positive sera (one sample for IgG and one sample for IgM) were two-fold serially diluted and their total protein concentration quantified by a Pierce™ BCA Protein-Assay-Kit (Thermo Fisher Scientific™, Waltham, MA, USA; 23225), following the manufacturer’s instructions. Later, each dilution point was previously analyzed by IFAT, until the fluorescence signal was no longer detected by microscope reading; afterwards, each dilution point was examined by Chorus Q Fever Phase II IgG and IgM kits to determine the OD. Finally, results obtained by IFAT and Chorus were compared.

#### 5.7.2. Analytical Specificity

ASp was determined by means of the detection of the cross-reactivity profile, by using a total of 66 sera related to five groups, respectively, from 10 patients clinically affected by Q fever and positive to *C. burnetii* (IgG and IgM) by IFAT (positive controls), 10 sera from healthy donors (negative controls), and 46 serum samples from patients without symptoms related to Q fever and that tested negative for the presence of antibodies versus *C. burnetii* (IgG and IgM) by IFAT but that were serologically positive for other pathogens. In particular, nine sera were IgG positive and six sera were IgM positive for *Bartonella* spp., five sera were IgG positive and five sera were IgM positive for *Rickettsia* spp., eight sera were IgG positive and 13 sera were IgM positive for *Chlamydia* spp., all of which were analyzed by Chorus Q Fever Phase II IgG and IgM kits. The ASp values were calculated by using a 2 × 2 contingency table on an Excel spreadsheet.

#### 5.7.3. Diagnostic Accuracy: Diagnostic Sensitivity, Diagnostic Specificity, Positive Predictive Value, and Negative Predictive Value

Diagnostic accuracy, diagnostic sensitivity (DSe) and diagnostic specificity (DSp) were tested by means of a statistically significant number of sera. An acceptable number of samples (n) was calculated using the formula *n* = {(4 × DSe × (1 − DSe))/e2}, considering that (e) was the amount of error allowed [49]. Since we assumed a DSe corresponding to 95%, the number of sera to analyze required at least 76 positive sera. Therefore, 340 sera samples (Table 7) from two groups of different subjects were analyzed by the Chorus system: a group of patients symptomatic for Q fever and who tested positive by IFAT corresponded to 101 samples for IgG and 63 for IgM (with a total of 164 samples) and a group of patients that were clinically healthy and tested negative by IFAT corresponded to 77 samples for IgG and 99 for IgM (a total of 176 samples). Samples were tested by the Chorus Q Fever Phase II IgG kit and by the Chorus Q Fever Phase II IgM kit. DSe, DSp, PPV, and NPV values were calculated by using a 2 × 2 contingency table on an Excel spreadsheet.

## Figures and Tables

**Table 1:** Operator performance: comparison between the results expected by clinical and indirect immunofluorescent antibody test (IFAT) findings with those obtained by the duplicate analysis by Chorus Q Fever Kits.

Chorus Q Fever IgG							
No. of Samples	Clinical/IFAT		No. of Samples	1st Analysis		2nd Analysis	
14	Positive		13	Positive		Positive	
6	Negative		6	Negative		Negative	
			1	Doubt		Doubt	
Total 20			Total 20				
**Chorus Q Fever IgM**							
**No. of Samples**	**Clinical Findings**	**IFAT Results**		**No. of Samples**	**1st Analysis**		**2nd Analysis**
6	Positive	Positive		5	Positive		Positive
14	Negative	Negative		1	Positive		Doubt
				11	Negative		Negative
				1	Negative		Doubt
				2	Doubt		Doubt
Total 20				Total 20			

**Table 2:** Repeatability and accuracy evaluation of Chorus Q Fever Kit: Cohen’s K values (K) According to Byrt [35].

		Chorus Q Fever IgG		Chorus Q Fever IgM
**Repeatability**	Excellent	K = 1.00	Very good	K = 0.88
**Accuracy**	Very good	K = 0.89	Excellent	K = 0.94

**Table 3:** Analytical sensitivity evaluation.

Chorus Q Fever IgG				
Dilution	IFAT	Chorus IgG	OD *	Total Proteins ** (µg/mL)
Not diluted	Positive	Positive	4.4	>2000
1:2	Positive	Positive	2.7	>2000
1:4	Positive	Positive	2.1	>2000
1:8	Positive	Doubt	1.1	>2000
1:16	Positive	Negative	0.5	>2000
1:32	Doubt	Negative	0.3	>2000
1:64	Negative	Negative	0.2	1146
1:128	Negative	Negative	0.1	620
1:256	Negative	Negative	0.1	261
1:512	Negative	Negative	0.0	170
1:1024	Negative	Negative	0.0	80
**Chorus Q Fever IgM**				
**Dilution**	**IFAT**	**Chorus IgM**	OD *	Total Proteins ** (µg/mL)
Not diluted	Positive	Positive	1.5	>2000
1:2	Positive	Doubt	0.8	>2000
1:4	Negative	Negative	0.5	>2000
1:8	Negative	Negative	0.4	>2000
1:16	Negative	Negative	0.2	>2000
1:32	Negative	Negative	0.2	>2000
1:64	Negative	Negative	0.2	1258
1:128	Negative	Negative	0.0	695
1:256	Negative	Negative	0.0	405
1:512	Negative	Negative	0.0	208
1:1024	Negative	Negative	0.0	98

* OD: Optical density; ** total protein quantified by Pierce™ BCA Protein-Assay-Kit (Thermo Fisher Scientific™, Waltham, MA, USA; 23225); positive samples: OD > 1.2; negative samples: OD < 0.8; Doubt samples: OD between 0.8 and 1.2.

**Table 4:** Analytical specificity of Chorus Q fever related to *Rickettsia* spp., *Bartonella* spp., and *Chlamydia* spp.

*Coxiella burnetii*					
Chorus Q Fever IgG			Chorus Q Fever IgM		
No. of Samples	clinical/IFAT	Chorus	No. of Samples	Clinical/IFAT	Chorus
5	Pos	Pos	5	Pos	Pos
5	Neg	Neg	5	Neg	Neg
***Rickettsia* spp.**					
**Chorus Q Fever IgG**			**Chorus Q Fever IgM**		
**No. of Samples**	**clinical/IFAT**	**Chorus**	**No. of Samples**	**Clinical/IFAT**	**Chorus**
5	Neg	Neg	5	Neg	Neg
***Bartonella* spp.**					
**Chorus Q Fever IgG**			**Chorus Q Fever IgM**		
**No. of Samples**	**clinical/IFAT**	**Chorus**	**No. of Samples**	**Clinical/IFAT**	**Chorus**
9	Neg	Neg	6	Neg	Neg
***Chlamydia* spp.**					
**Chorus Q Fever IgG**			**Chorus Q Fever IgM**		
**No. of Samples**	**clinical/IFAT**	**Chorus**	**No. of Samples**	**Clinical/IFAT**	**Chorus**
7	Neg	Neg	9	Neg	Neg
1	Neg	Doubt	1	Neg	Pos
			3	Neg	Doubt

**Table 5:** Diagnostic sensitivity and specificity of Chorus Q fever IgG.

Clinical/IFAT					
		Positive	Negative	Total	
**Chorus Q fever IgG Test Results**	**Positive**	(TP)	(FP)	(TP + FP)	PPV [TP/(TP + FP)]
		98	6	104	94.2
	**Negative**	(FN)	(TN)	(FN + TN)	NPV [TN/(TN + FN)]
		3	71	74	95.9
	**Total**	101	77	178	
		(TP + FN)	(FP + TN)	(TP + FP + FN + TN)	
		DSe	DSp		
		97.0	92.2		
		TP/(TP + FN)	TN/(TN + FP)		

DSe: diagnostic sensitivity; DSp: diagnostic specificity; FN: false negative; FP: false positive; NPV: negative predictive value; PPV: positive predictive value; TN: true negative; TP: true positive.

**Table 6:** Diagnostic sensitivity and specificity Chorus Q fever IgM.

		Clinical/IFAT			
		Positive	Negative	Total	
**Chorus Q fever IgM Test Results**	**Positive**	(TP)	(FP)	(TP + FP)	PPV [TP/(TP + FP)]
		60	6	66	90.9
	**Negative**	(FN)	(TN)	(FN + TN)	NPV [TN/(TN + FN)]
		3	93	96	96.8
	**Total**	63	99	162	
		(TP + FN)	(FP + TN)	(TP + FP + FN + TN)	
		DSe	DSp		
		95.2	93.9		
		TP/(TP + FN)	TN/(TN + FP)		

DSe: diagnostic sensitivity; DSp: diagnostic specificity; FN: false negative; FP: false positive; NPV: negative predictive value; PPV: positive predictive value; TN: true negative; TP: true positive.

**Table 7:** Description of the samples involved in the study.

Positive Sera			Negative Sera		
**Chorus IgG**	**Chorus IgM**		**Chorus IgG**	**Chorus IgM**	
101	63	**Total 164**	77	99	Total 176 *

* 46 negative sera belonged to patients affected by other pathogens: *Chlamydia* spp. (21), *Rickettsia* spp. (*n* = 10) and *Bartonella* spp. (*n* = 15).
